# Immunotherapy for Platinum-Resistant Ovarian Cancer as a Glimmer of Hope

**DOI:** 10.3390/cells14130995

**Published:** 2025-06-29

**Authors:** Jan Brancewicz, Irena Barbara Padzińska-Pruszyńska, Małgorzata Kubiak, Paulina Kucharzewska

**Affiliations:** 1 Cellis AG, Bleicherweg 45, 8002 Zurich, Switzerland; 2 Center of Cellular Immunotherapies, Warsaw University of Life Sciences, Building 23, Level 0, Laboratory Number 0135, 8 Ciszewskiego St., 02-786 Warsaw, Poland; irena_pruszynska1@sggw.edu.pl (I.B.P.-P.); malgorzata_kubiak1@sggw.edu.pl (M.K.)

**Keywords:** ovarian cancer, cancer immunotherapy, cell-based therapies, tumor-associated macrophages, tumor microenvironment

## Abstract

Ovarian cancer is a leading cause of cancer-related deaths among women, with platinum resistance posing a significant challenge. Conventional therapies often fail in these cases, highlighting the urgent need for novel treatment approaches. Immunotherapy has emerged as a promising strategy, offering renewed hope for patients with platinum-resistant ovarian cancer (PROC). This review explores the current landscape of immunotherapies for PROC, discussing different approaches, their mechanisms of action, and the potential for overcoming the limitations of conventional treatments.

## 1. Introduction

Ovarian cancer (OC) is currently ranked as the eighth most commonly diagnosed cancer among women worldwide and continues to pose a serious threat to public health [1]. Despite its relatively lower incidence compared to other malignancies, OC accounts for a disproportionately high number of cancer-related deaths. As of 2024, an estimated 320,000 new cases and 210,000 deaths were attributed to OC globally, representing approximately 4.8% of all cancer-related mortality [1]. This big contrast between incidence and mortality is largely attributable to the advanced stage at which the disease is typically diagnosed. The asymptomatic nature of OC in its early stages frequently results in late-stage diagnoses, which in turn contribute to its poor prognosis and high mortality rate [2]. Notably, women diagnosed with stage I OC have a favorable 5-year survival rate of approximately 91%. In contrast, survival rates decline to about 72% for those with regional disease, confined to the pelvis (stage II), and decrease substantially to around 22% for those with the metastatic disease (stage III–IV) [2]. However, only about 21% of cases are diagnosed at these earlier, more treatable stages, underscoring the silent and insidious nature of early OC progression. This asymptomatic presentation contributes to diagnostic delays and remains a significant barrier to improving survival rates [1].

Risk factors for OC include genetic predisposition (e.g., *BRCA1/2* mutations), family history, nulliparity, early menarche, late menopause, and certain lifestyle factors such as obesity and smoking. OC primarily affects postmenopausal women, with the majority of cases diagnosed after the age of 50, and the peak incidence occurring between 55 and 64 years [3,4].

OC is classified into three major types based on the cell of origin and histopathological characteristics: epithelial (EOC), germ cell, and sex cord stromal OC. Non-epithelial OCs account for less than 10% of all OCs and generally have a more favorable prognosis [5]. EOC is the most prevalent form of OC, accounting for approximately 90% of all ovarian malignancies. It originates from the epithelial cells lining the outer surface of the ovary, fallopian tube, or peritoneum [6]. EOC is classified into several histological subtypes with distinct molecular characteristics, prognosis, and treatment responses. These subtypes include serous, mucinous, endometrioid, clear-cell, and transitional cell carcinomas [7]. Serous carcinomas are further divided into high-grade (HGSCs) and low-grade serous carcinomas (LGSCs), with HGSCs constituting about 75% of all epithelial subtypes and being recognized as the most aggressive and lethal forms of OC [8,9].

Despite advancements in surgical and chemotherapeutic approaches, recurrence is common, and long-term survival remains poor. Improved early detection strategies and personalized treatment approaches are urgently needed to enhance outcomes for women affected by this disease.

HGSC typically responds well to initial platinum-based chemotherapy; however, the majority of patients (approximately 75–80%) with advanced-stage disease eventually develop recurrence, and some of them developed chemoresistance [10]. This condition, termed platinum-resistant ovarian cancer (PROC), severely limits therapeutic options and contributes to poor long-term survival outcomes [11]. Even among patients diagnosed at an early stage, approximately 20% will experience recurrence, although the rates of resistance are somewhat lower in this group [12]. The development of platinum resistance is a critical point in the clinical management of OC and is associated with a significantly poorer prognosis [13,14].

The predominance of late-stage diagnoses, high recurrence rates, and the rapid onset of chemoresistance in OC highlight the critical need for innovative approaches to early detection, effective predictive biomarkers, and novel therapeutic strategies. The current standard treatments, while initially effective in many cases, fail to provide durable responses for the majority of patients, particularly those with platinum-resistant disease [11]. As such, improving long-term outcomes demands a paradigm shift in diagnostics and therapeutics. Emerging treatment modalities, especially targeted therapies and immunotherapies, hold substantial promise. Immunotherapy, in particular, has gained attention as a viable option that leverages the body’s immune system to recognize and eliminate cancer cells [15]. A range of immunotherapeutic strategies, including next-generation immune checkpoint inhibitors (ICIs), chimeric antigen receptor (CAR)-based therapies, and tumor vaccines, are currently under active investigation in the context of OC. These emerging modalities have the potential to revolutionize the therapeutic landscape by addressing the limitations of conventional treatments. Their successful integration into clinical practice may offer more effective and durable options for patients, particularly those with treatment-resistant disease and otherwise limited prospects for long-term survival.

In this review, we will explore the definition and clinical characteristics of primary resistance in OC patients. We will examine key factors contributing to such resistance, evaluate current therapeutic strategies, and discuss future immunotherapy approaches aimed at improving survival rates and quality of life for affected women.

## 2. Platinum-Resistant Ovarian Cancer

PROC is a clinically challenging subset of recurrent ovarian cancer (ROC), defined by disease progression within six months following the completion of platinum-based chemotherapy. It is associated with poor treatment response, limited therapeutic options, and significantly reduced overall survival (OS) [16]. Approximately 22–28% of women with ROC fall into this category, making PROC a major contributor to OC-related mortality [17].

ROC typically arises from residual cancer cells that were not fully eliminated during initial treatment. The likelihood of recurrence is influenced by several factors, including the stage at diagnosis, molecular composition of the tumor, and effectiveness of the initial therapy. While ROC is rarely curable, treatment primarily focuses on symptom control and prolonging survival, with a median survival time after recurrence of approximately two years [18]. However, individual outcomes can vary significantly based on patient- and disease-specific factors.

PROC can be further classified into two subtypes based on the timing and nature of resistance development. Primary platinum-resistant ovarian cancer (PPROC) is characterized by intrinsic resistance, with recurrence occurring within six months of completing first-line platinum-based chemotherapy. It accounts for approximately 25% of all OC cases and is associated with particularly poor outcomes, with median OS ranging from 9 to 17 months. Secondary platinum-resistant ovarian cancer (SPROC), on the other hand, develops after an initial response to platinum therapy, followed by relapse within six months of subsequent treatment. While prognosis in SPROC is generally more favorable than in PPROC, outcomes remain suboptimal [17,18].

Resistance to platinum compounds remains one of the most significant obstacles in the treatment of OC, affecting up to 85% of patients who initially respond well to platinum-based regimens [18]. Most patients with initial sensitivity eventually develop secondary resistance over the course of multiple relapses, often resulting in progressively shorter progression-free survival (PFS) intervals. Traditionally, platinum resistance has been defined by the interval between completion of platinum-based therapy and disease recurrence: patients relapsing within six months are considered platinum-resistant, while those relapsing after six months are termed platinum-sensitive [2,18].

However, this binary classification is becoming increasingly limited in clinical relevance, particularly with the advent of agents like bevacizumab and olaparib, which can prolong progression-free intervals. Notably, in some cases, patients who relapse after more than six months may still exhibit poor responses to platinum rechallenge (response rates < 50%), whereas those who relapse earlier may still derive benefit from platinum-based combinations [2].

Management of recurrent or platinum-resistant disease is multifaceted and must be tailored to individual patient factors, including time since last treatment, previous therapies, and overall health status. Therapeutic options may include chemotherapy, targeted therapies, surgery, and hormonal approaches, depending on the biological characteristics of the disease and treatment history.

## 3. Molecular Mechanisms of Platinum Resistance

**Figure 1:**
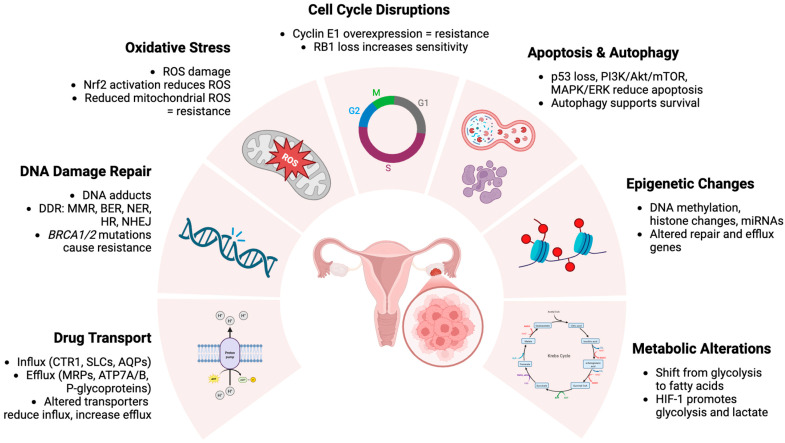
Factors driving the development of platinum resistance in OC cells. Created using BioRender.com.

The development of resistance to platinum therapy involves a complex interplay of multiple factors such as altered drug uptake and efflux, DNA repair enhancement, mutations in genes involved in cell death pathways, oxidative stress, cell cycle disruption, epigenetic changes, or metabolic alteration (Figure 1), often resulting in the activation of multiple resistance mechanisms simultaneously.

### 3.1. Alterations in Drug Influx and Efflux Pathways

Platinum-based drugs enter cells either by passive diffusion or using membrane transporters [19], and cancer cells are often equipped with protective mechanisms that allow them to expel these drugs. Cisplatin is imported by the members of the solute carrier superfamily such as copper transport proteins (CTRs), solute carrier transporters (SLCs), and aquaporins (AQPs), and is exported by efflux ATPases like MRPs and ATP7A/B and P-glycoproteins, also known as multidrug resistance proteins (MDRs) [20,21,22,23]. Alterations in the expression of these transporters can lead to reduced influx and/or increased efflux of cisplatin, which further contribute to chemoresistance in several types of cancer, including OC. Multiple studies have shown that CTR1 knockout leads to enhanced resistance to cisplatin, while its overexpression causes an increase in drug accumulation and, in most cases, higher cisplatin sensitivity [20,24,25,26,27]. Inhibition of MDRs may be a good strategy to counteract drug resistance. One example of P-glycoprotein modulators is valspodar, which has shown promising results in clinical trials for overcoming OC resistance [28,29].

### 3.2. DNA Damage Repair Enhancement

Platinum-based agents exert their cytotoxic effects by forming DNA adducts, and cell sensitivity to these drugs is influenced by the capacity to recognize and repair this damage through various DNA damage response (DDR) pathways [30,31]. Key DDR pathways include mismatch repair (MMR), base excision repair (BER), nucleotide excision repair (NER), homologous repair (HR), and non-homologous end joining (NHEJ). Abnormal activity in these pathways can lead to increased DNA repair capacity, contributing to platinum resistance [30,31,32]. To illustrate, upregulation of NER proteins like ERCC1 and XPA can enhance resistance by efficiently removing platinum adducts [33]. Interestingly, mutations in *BRCA1/2* genes, which are crucial for the HR pathway [34], play a dual role in OC treatment. They can initially increase sensitivity to platinum agents; however, a prolonged exposure to these drugs can cause the development of resistance through reversion mutations that restore HR proficiency [35,36] (~50% of platinum-resistant tumors with initial *BRCA1/2* mutation [37,38]).

### 3.3. Reactive Oxygen Species Regulations and Oxidative Stress

Platinum drugs induce an increase in reactive oxygen species (ROS) levels, causing DNA damage that may lead to genomic instability, senescence, or apoptosis. However, tumor cells can avoid it by activating Nrf2, a transcription factor involved in the regulation of antioxidant response, which is associated with resistance to cisplatin [39]. Additionally, the inhibition of Nrf2 has been shown to sensitize cancer cells to this drug by enhancing ROS-mediated damage and consequently, promoting cell death [40]. Recent studies have demonstrated that cisplatin-induced ROS upregulate DCTPP1, a nucleotide triphosphate pyrophosphatase that mitigates oxidative DNA damage by stabilizing dNTP pools and activating the Nrf2-mediated antioxidant pathway, thereby reducing apoptosis and enhancing chemoresistance in OC [41,42].

Mitochondria, key regulators of intracellular ROS generation, also play an important role in determining the effectiveness of the cytotoxic effects of platinum-based chemotherapy. In HGSC, resistance to cisplatin has been linked to reduced mitochondrial ROS production. Cisplatin targets not only nuclear but also mitochondrial DNA, and its interaction with the latter can lead to impaired mitochondrial function, decreased ROS generation, and consequently reduced ROS-induced DNA damage and cell death [43,44].

### 3.4. Cell Cycle Disruptions

Alterations in the expression of cell cycle regulators such as cell cycle regulator cyclin E1 (CCNE1) and retinoblastoma protein 1 (RB1) have also been shown to influence the platinum sensitivity of OC. *CCNE1* gene amplification is linked to cisplatin resistance, as cyclin E1 interacts with and activates CDK2, a crucial regulator of DNA replication initiation and G1/S phase progression [45,46]. Overexpression of cyclin E1 results in premature CDK2 activation, initiating DNA replication before pre-replication complexes at origins are adequately licensed [47]. This unscheduled origin firing induces replication stress, leading to DNA damage and genomic instability [48,49,50]. These alterations allow cancer cells to evade apoptosis and adapt to DNA damage, thereby contributing to the development of cisplatin resistance. As a result, cyclin E1-overexpressing cells are able to survive and proliferate despite the presence of DNA-damaging agents such as cisplatin, significantly reducing the efficacy of the treatment [51,52]. In contrast, a loss of RB1, a key tumor suppressor involved in the regulation of transition from G1 to S, correlates with higher platinum sensitivity and improved survival outcomes in HGSC patients [53].

### 3.5. Mutations in Apoptotic Pathways and Autophagy

Cisplatin resistance in OC can also be driven by molecular changes that lead to the disruption of apoptotic pathways, particularly through p53 dysfunction [54], and the activation of compensatory survival signaling cascades such as PI3K/Akt/mTOR [55,56] or MAPK/ERK [57,58]. Approximately 40–80% of EOCs harbor *TP53* mutations, which impair the p53-mediated activation of pro-apoptotic genes and reduce sensitivity to platinum-induced DNA damage [54,59].

OC cisplatin resistance can be further explained by ERK-mediated autophagy, which promotes cell survival during cisplatin exposure [58], and by collagen type XI alpha 1-driven upregulation of three apoptosis inhibitors (XIAP, BIRC2, BIRC3) via Src-PI3K/Akt-NF-κB signaling [60]. These mechanisms collectively inhibit caspase activation and mitochondrial apoptosis, thereby contributing to OC cells’ resistance to cisplatin-induced cytotoxicity.

### 3.6. Epigenetic Changes

Epigenetic modifications are also associated with the development of platinum resistance in OC. One of the key epigenetic mechanisms, DNA methylation, plays an important role in OC chemoresistance as platinum-resistant samples often exhibit higher levels of hypermethylation in comparison to the sensitive ones [61]. Interestingly, hypomethylation can also influence resistance. For example, decreased expression of msh homebox 1 transcription factor caused by DNA hypomethylation has been linked to cisplatin resistance [62,63].

Apart from DNA methylation, other epigenetic modifications have also been shown to contribute to OC platinum resistance. For instance, overexpression of histone deacetylases such as histone deacetylase 1/10 and sirtuin 5 has been observed in platinum-resistant OC [64,65]. Additionally, dysregulation of microRNAs (miRNAs) such as miR-139 and miR-211 can modulate platinum sensitivity by targeting genes involved in DNA repair and drug efflux [66,67]. These miRNAs can enhance sensitivity by inhibiting pathways that confer resistance, highlighting their potential as therapeutic targets.

### 3.7. Metabolic Alterations

Metabolic reprogramming, including changes in glucose, glutamine, lipid, and mitochondrial metabolism, plays a significant role in cisplatin resistance in OC [68,69,70,71]. Resistant cells often shift from glucose-dependent glycolysis to fatty acid uptake and β-oxidation, offering an alternative energy source that supports survival under cisplatin-induced oxidative stress [72]. Interestingly, the impact of glucose metabolism alterations may vary, with some studies reporting increased glycolysis in cisplatin-resistant cells, while others highlight a shift toward oxidative phosphorylation [69,73].

Hypoxia-induced HIF-1 activation further contributes to this metabolic plasticity by promoting glycolysis and lactate production, thus facilitating adaptation to low oxygen levels. This metabolic shift allows tumor cells to survive in a hypoxic microenvironment, which is often present in solid tumors, including OC. Notably, inhibiting HIF-1α has been shown to enhance cisplatin sensitivity by increasing ROS production, and, consequently, disrupting redox homeostasis and promoting apoptosis [74].

## 4. Current Standard of Care for Recurrent Ovarian Cancer

Chemotherapy is a primary treatment option for ROC, often used to increase survival time and relieve symptoms [75]. The choice of chemotherapy is based on the platinum-free interval (PFI), which is the time between the completion of the last platinum-based treatment and the recurrence. This is because the PFI correlates with PFS, OS, and response to subsequent treatment. For patients with platinum-sensitive recurrent ovarian cancer (PSROC), defined as recurrence occurring more than six months after the last platinum-based therapy, platinum-based regimens are often reinitiated [75]. Common combinations include carboplatin with paclitaxel, gemcitabine, or pegylated liposomal doxorubicin (PLD) [76,77,78]. In cases of PROC, defined as the ROC with recurrence occurring within 6 months of the last platinum treatment, non-platinum agents such as weekly paclitaxel, PLD, gemcitabine, or topotecan are used [79,80,81].

Targeted therapies have become increasingly important in managing ROC. Bevacizumab is used in combination with chemotherapy to enhance efficacy and improve symptom control for both PROC and PSROC [16,82,83,84]. Additionally, poly (ADP-ribose) polymerase inhibitors (PARPi; such as olaparib, niraparib, and rucaparib) are recommended as maintenance therapy following response to platinum-based chemotherapy, regardless of *BRCA* status [85,86,87,88,89]. One of the newest treatment options for PROC is Mirvetuximab soravtansine-gynx (MIRV), an antibody–drug conjugate directed against FRα, the effectiveness of which has been demonstrated in combating PROC in a phase 3 clinical trial [90].

An emerging therapy for PROC is relacorilant, a selective glucocorticoid receptor antagonist. In the phase III ROSELLA trial, relacorilant combined with nab-paclitaxel significantly improved outcomes, with a median PFS of 5.6 months vs. 3.8 months and median OS of 16.3 months vs. 11.4 months compared to chemotherapy alone. These results highlight its potential role in future treatment strategies for PROC [91].

Surgery can be considered for selected patients with ROC, particularly those with PSROC and favorable prognostic factors [92]. This procedure aims to achieve secondary cytoreduction in cases with a single site of recurrence, which has been associated with improved survival outcomes or to relieve symptoms [93,94,95].

Hormonal therapies using drugs like tamoxifen (an estrogen inhibitor) and aromatase inhibitors (AIs), such as letrozole and anastrozole, are an option for patients with hormone receptor-positive ROC, especially those with platinum-resistant tumors, either as maintenance therapy or when chemotherapy is not well tolerated, due to age, comorbidities, or previous toxicities [96,97,98,99,100]. This approach is often chosen due to its favorable side effect profile and oral administration. Further research and clinical trials are ongoing to refine the role of hormonal therapies in OC treatment. The standard of care for ROC is summarized in Table 1.

## 5. The Rise of Cancer Immunotherapy

**Figure 2:**
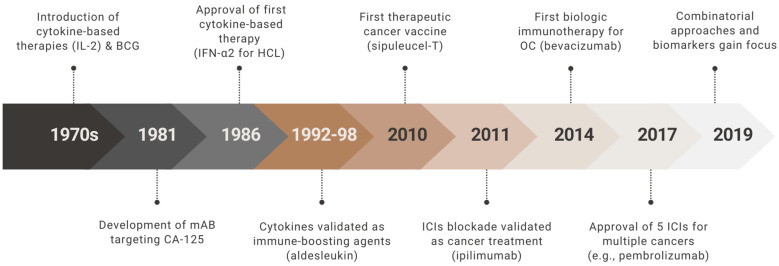
Timeline of key milestones in the rise of OC immunotherapy.

The development of cancer immunotherapy has followed a long and evolving path, with key milestones that have progressively shaped its clinical utility (Figure 2). In OC, and particularly in PROC, the translation of these advances has been slower than in other malignancies, but recent years have brought growing momentum.

The conceptual foundation of immunotherapy was laid in the 1950s, when Burnet and Thomas proposed the cancer immunosurveillance hypothesis—suggesting that the immune system plays a role in detecting and eliminating malignant cells [101]. This idea gained further support in the 1980s with the emergence of cytokine-based therapies such as interleukin-2 (IL-2) [102,103] and interferon-α [104], as well as the development of adoptive cell transfer (ACT) [105]. Around the same time, the first significant breakthrough in OC immunology occurred with the discovery of the OC125 monoclonal antibody, which recognized cancer antigen 125 (CA-125), a circulating epitope of the transmembrane mucin MUC16, and laid the groundwork for both diagnostic and therapeutic targeting [106].

The 1990s marked a pivotal era in cancer immunotherapy, with the identification of tumor-associated antigens (TAAs) like MAGE-1 [107], and key insights into immune checkpoint biology. The discovery of cytotoxic T-cell antigen 4 (CTLA-4) [108] and programmed death-1 (PD-1) [109] as inhibitory receptors on T cells provided a mechanistic explanation for immune evasion by tumors. Meanwhile, the first immunotherapy trials in OC began, including vaccine candidates such as abagovomab (targeting CA-125) [110] and early attempts at tumor-infiltrating lymphocyte (TIL) therapy [111]. While these studies demonstrated feasibility and safety, clinical benefit remained limited.

In the 2000s, the potential of immune checkpoint blockade became evident with the success of CTLA-4 [112] and PD-1 [113,114] inhibitors in melanoma. Although OC was less responsive, the biological rationale remained compelling—especially given the presence of TILs and the observation that tumors with homologous recombination deficiency (HRD) or *BRCA* mutations often exhibit higher neoantigen loads [113]. Around the same time, bevacizumab, an anti-angiogenic monoclonal antibody with immunomodulatory effects, became the first biologic agent approved for ROC, offering modest but significant gains in PFS [16].

The 2010s saw large-scale testing of immune checkpoint inhibitors (ICIs) in OC. Single-agent PD-1/PD-L1 inhibitors such as nivolumab and pembrolizumab yielded modest objective response rates (8–15%) and disease control in roughly 40–50% of patients with PROC [115,116], with responses lasting 8–13 months in some cases [117]. These results underscored the immunosuppressive nature of the ovarian tumor microenvironment (TME), characterized by regulatory T cells (Tregs), myeloid-derived suppressor cells (MDSCs), and tumor-associated macrophages (TAMs) [118]. In response, research turned toward combination strategies. Trials such as NRG-GY003 demonstrated improved efficacy with dual checkpoint blockade (nivolumab + ipilimumab) [117], and the MEDIOLA trial explored the combination of durvalumab with the PARP inhibitor olaparib in *BRCA*-mutant OC [119].

Entering the 2020s, new immunotherapeutic strategies began to emerge. CAR-T cells targeting mesothelin, MUC16, and folate receptor-α (FRα) entered clinical trials [120], alongside first-in-human studies of CAR-macrophages (CAR-M), which aim to overcome the limitations of T-cell infiltration in solid tumors [121]. TIL therapy is also being adapted for OC, with early-phase trials exploring its feasibility and durability [122]. At the same time, bispecific T-cell engagers, CD47 inhibitors, STING agonists, and agents targeting LAG-3 or TIGIT are under investigation to address resistance to PD-1-based therapies.

Therapeutic cancer vaccines are also experiencing a resurgence. Oregovomab, an anti-CA-125 antibody-based vaccine, showed encouraging results in a phase II trial when combined with chemotherapy, and is currently being evaluated in the phase III FLORA-5 trial [123]. Neoantigen-targeted mRNA vaccines—modeled after recent successes in melanoma—are entering early-phase trials in OC [124].

Despite the lack of an FDA- or EMA-approved immunotherapy for PROC as of 2025, a growing number of trials are underway, many of which aim to personalize treatment based on molecular markers such as *BRCA* status, HRD, or programmed death-ligand 1 (PD-L1) expression. The cumulative progress from early immunosurveillance theory to checkpoint inhibition, cellular therapy, and rational combination strategies reflects a paradigm shift in the management of OC. While challenges remain, immunotherapy continues to offer hope for more effective, durable, and personalized treatment approaches for patients with platinum-resistant disease.

## 6. Diverse Immunotherapy Strategies

The convergence of checkpoint inhibitors, cellular therapies, cancer vaccines, and immune-modulating agents represents a comprehensive effort to overcome the challenges of immunotherapy in OC. While immune checkpoint blockade alone has shown limited efficacy, ongoing studies are refining combination approaches, identifying novel immune targets, and optimizing personalized immunotherapies to improve clinical outcomes.

**Figure 3:**
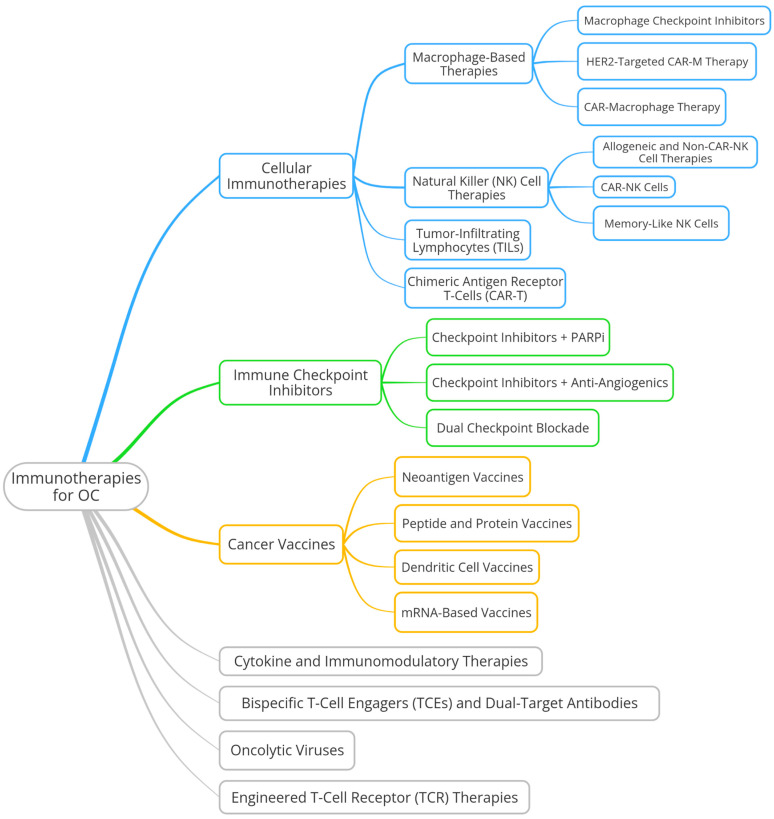
Most promising immunotherapies for OC in development.

Building on this foundation, the next sections will explore the diverse immunotherapeutic strategies currently being investigated in OC (Figure 3), examining their mechanisms, clinical development, and potential for integration into standard treatment paradigms.

### 6.1. Cellular Immunotherapies

Cell-based immunotherapies represent a cutting-edge approach for PROC, designed to enhance the ability of immune cells to recognize and eliminate tumor cells. These strategies leverage engineered T cells, TILs, natural killer cells, and macrophages, offering promising avenues to overcome resistance and improve patient outcomes.

#### 6.1.1. Chimeric Antigen Receptor T Cell (CAR-T)

CAR-T therapy, which has demonstrated remarkable success in hematologic malignancies, is being adapted for OC through targeted strategies against TAAs. Early-phase clinical trials since 2021 have focused on CAR-T cells engineered to recognize mesothelin, FRα, MUC16 (CA-125), B7-H3, and other OC antigens [125].

A notable advancement is the first-in-human CAR-T therapy targeting the follicle-stimulating hormone receptor (FSHR), an antigen aberrantly expressed on OC cells. In the ongoing phase I trial (NCT05316129) at Moffitt Cancer Center, autologous T cells are engineered with a chimeric endocrine receptor to selectively target FSHR-positive tumors. Interim data indicate that FSHR CAR-T therapy is well tolerated, with no dose-limiting toxicities observed [126]. One patient in the lowest-dose cohort experienced disease stabilization and minor tumor regression lasting over a year, suggesting potential efficacy in heavily pretreated OC patients. The trial is now evaluating higher-dose cohorts and comparing intraperitoneal (IP) vs. intravenous (IV) CAR-T delivery to optimize tumor targeting [127].

Beyond FSHR, mesothelin-directed CAR-T therapy has demonstrated preliminary activity in OC [128]. In a City of Hope trial, CAR-T cells against TAG72, another OC antigen, successfully eradicated tumors in preclinical models and have entered a phase I trial for advanced OC [129]. However, early-generation mesothelin CAR-T trials in OC yielded only partial responses, with efficacy limited by CAR-T exhaustion and the immunosuppressive TME [128].

To address these barriers, researchers are developing “armored” CAR-T cells designed to secrete cytokines such as IL-12, which counteract PD-1/PD-L1-mediated suppression. Fourth-generation CAR-T cells expressing IL-12 have shown improved tumor control in OC models, suggesting a strategy to overcome immune evasion [130]. Another innovative approach is BioNTech’s BNT211 therapy, which pairs CLDN6-specific CAR-T cells with an IVT mRNA “CARVac” vaccine to enhance CAR-T persistence and efficacy. In a phase I/II trial for solid tumors, including OC, BNT211 achieved a 45% ORR, with disease control in 74% of patients. However, over half of the patients experienced cytokine release syndrome (CRS), a known CAR-T side effect, highlighting the need for further refinement of toxicity management in solid tumors [131].

These advancements demonstrate that CAR-T therapy can mediate anti-tumor activity in OC when optimized for persistence, immune evasion, and tumor penetration. Ongoing research is focused on next-generation CAR-T strategies, including combinatorial therapies with ICIs and TME modulators, to enhance efficacy in solid tumors.

#### 6.1.2. Tumor-Infiltrating Lymphocytes (TILs)

TIL therapy is an ACT strategy in which patient-derived T cells are isolated from tumors, expanded ex vivo, and reinfused to enhance anti-tumor immunity [132]. This approach has demonstrated remarkable success in melanoma [132] and is now being investigated for PROC.

Early pilot studies have shown encouraging results. In one cohort of seven OC patients treated with TILs, an ORR of 71% was reported, including one complete response and four partial responses. Another cohort combining TIL infusion with low-dose cyclophosphamide intended to deplete regulatory immune cells and enhance T-cell engraftment also demonstrated promising activity [132].

Building on these findings, Iovance Biotherapeutics has been developing LN-145, an autologous TIL product, for ROC. While detailed efficacy data for OC are pending, Iovance’s parallel studies in cervical cancer demonstrated a 44% response rate, providing a strong rationale for continued investigation in gynecologic malignancies [133]. Ongoing phase II trials are currently assessing the efficacy of LN-145 in PROC [134].

A key advantage of TIL therapy is its potential for durable responses. In some patients, TIL or CAR-T therapies have led to long-term remissions measured in years, suggesting that TIL persistence and sustained immune surveillance may contribute to prolonged tumor control. Efforts to further optimize TIL therapy for OC include combining TILs with IL-2 support to enhance T-cell survival post-infusion, integrating checkpoint inhibitors (e.g., PD-1 blockade) to counteract tumor-mediated immunosuppression, and improving patient selection criteria, such as identifying individuals with pre-existing TILs or PD-L1 expression, who may be more likely to benefit from therapy [132].

As TIL manufacturing techniques advance and patient selection strategies improve, TIL therapy could emerge as a viable option for PROC, offering durable anti-tumor responses in a subset of patients with limited treatment alternatives.

#### 6.1.3. Natural Killer (NK)-Cell Therapies

NK cells are innate immune effectors capable of eliminating tumor cells without prior antigen sensitization, making them an attractive immunotherapeutic option for PROC [135]. Several NK-based strategies, including memory-like NK cells, CAR-engineered NK cells, and allogeneic NK-cell infusions, are currently being investigated to enhance anti-tumor responses in OC.

##### Memory-like NK Cells

Memory-like NK cells, which exhibit enhanced persistence and recall responses, are generated by cytokine pre-activation ex vivo before infusion [136]. In late 2024, Dana-Farber Cancer Institute initiated a phase I trial evaluating cytokine-induced memory-like NK cells in recurrent PROC. Preclinical models have demonstrated that these pre-activated NK cells generate strong anti-tumor responses against OC, and this ongoing trial aims to assess safety and early clinical efficacy in patients with limited treatment options [137].

##### CAR-NK Cells

CAR-modified NK cells combine NK-mediated cytotoxicity with targeted tumor recognition, enhancing specificity and persistence. Unlike CAR-T cells, CAR-NK cells do not induce graft-versus-host disease (GVHD), allowing them to be derived from healthy donors or cord blood, creating an “off-the-shelf” allogeneic therapy [138].

At MD Anderson Cancer Center, a phase I/II trial (NCT05922930) is evaluating TROP2-targeted CAR-NK therapy in platinum-resistant HGSC. This therapy utilizes cord blood-derived NK cells transduced with a CAR targeting TROP2 and an IL-15 support gene, designed for IP delivery, to enhance tumor targeting in the peritoneal cavity. Preclinical data show that TROP2 is frequently overexpressed in OC, and CAR-NK cells armed with cytokine support (IL-15) exhibit potent tumor cell killing [139].

Another CAR-NK therapy (NCT03692637) in China is targeting mesothelin, a well-established OC antigen. This approach uses NK-92 cells engineered with a mesothelin-specific CAR, and early reports suggest safety with preliminary anti-tumor activity in PROC patients [138].

##### Allogeneic and Non-CAR NK-Cell Therapies

NK-based therapies have also been tested without CAR engineering. Infusions of allogeneic NK cells or cord blood NK cells have resulted in transient disease stabilizations in some PROC patients. Additionally, NK cells combined with monoclonal antibodies leveraging antibody-dependent cellular cytotoxicity (ADCC) are under investigation as adjuncts to ICIs or chemotherapy to enhance tumor targeting [140].

NK-cell therapies provide a distinct immunotherapeutic approach to T-cell-based therapies, offering an alternative mechanism of tumor recognition and elimination. Strategies such as memory-like activation, CAR engineering, and cytokine support aim to improve NK-cell persistence and tumor infiltration in OC. Ongoing clinical trials will determine the potential of NK-based therapies as a viable treatment option for PROC.

#### 6.1.4. Macrophage-Based Therapies

TAMs are highly abundant in the OC microenvironment, where they contribute to immune suppression and tumor progression. Macrophage-based therapies aim to reprogram TAMs into tumor-fighting phenotypes or harness engineered macrophages to directly eliminate cancer cells.

##### CAR-Macrophages (CAR-M)

A breakthrough in macrophage-based immunotherapy is CAR-M, which are genetically engineered to recognize and phagocytose tumor cells upon CAR activation [141]. In 2024, Zhang et al. reported the first-in-human CAR-M trial in OC, treating two patients with recurrent disease using mesothelin-targeting CAR-M (SY001). These autologous CAR-M cells, administered intravenously, demonstrated a favorable safety profile, with no high-grade toxicities or CRS. Both patients achieved stable disease at 28 days post-infusion, though no tumor regressions were observed with a single dose. Importantly, CAR-M cells persisted in circulation, showing evidence of immune system engagement, including transient cytokine elevations [142].

To improve efficacy, researchers are exploring IP CAR-M delivery—a strategy expected to enhance local tumor targeting in peritoneal metastases. Additionally, CAR-M combinations with anti-PD-1 checkpoint inhibitors are being tested to further activate adaptive immunity [141].

##### HER2-Targeted CAR-M Therapy

A phase I trial at the University of Pennsylvania is evaluating human epidermal growth factor receptor 2 (HER2)-directed CAR-M (CT-0508) in solid tumors, including OC. Among 14 patients treated, the therapy was well tolerated, with no dose-limiting toxicities or severe immune-related events. The best response observed was stable disease in ~29% of patients, all of whom had HER2-overexpressing tumors. While tumor regression was not achieved, treatment led to macrophage infiltration and chemokine alterations, indicating biologic activity [121].

##### Macrophage Checkpoint Inhibitors

To further enhance macrophage-mediated tumor clearance, targeting macrophage-specific immune checkpoints is under investigation. CD47/SIRPα blockade disrupts the “don’t eat me” signal, enabling macrophages to phagocytose tumor cells. The CD47 inhibitor magrolimab has shown preclinical synergy with CAR-M therapy and opsonizing antibodies in OC models. Early-phase trials are now testing anti-CD47 agents in combination with CAR-M therapy and ICIs [143].

Another macrophage-directed strategy involves CSF-1 receptor (CSF1R) inhibition, which reduces the immunosuppressive TAM population within ovarian tumors. Small-molecule CSF1R inhibitors and monoclonal antibodies are being evaluated in PROC patients to shift the macrophage phenotype toward a pro-inflammatory, tumoricidal state [144].

Macrophage-based therapies represent a novel frontier in cellular immunotherapy for PROC, with CAR-M, CD47 blockade, and CSF1R inhibition offering complementary strategies to reprogram or enhance macrophage anti-tumor activity. While still in early clinical development, initial findings demonstrate feasibility, immune engagement, and disease stabilization, supporting continued exploration of macrophage-targeted immunotherapies as part of multi-modal treatment strategies for OC.

### 6.2. Immune Checkpoint Inhibitors

ICIs, which release inhibitory signals on T cells, were among the first immunotherapies tested in PROC. While PD-1 (nivolumab, pembrolizumab) and PD-L1 (atezolizumab, durvalumab, avelumab) inhibitors have shown modest single-agent activity, with response rates of 8–15%, OC’s immunologically “cold” TME has limited their effectiveness [145]. Recent efforts have shifted toward combination strategies and novel checkpoint targets to enhance immune activation and clinical benefit in PROC.

#### 6.2.1. Dual Checkpoint Blockade

Combining PD-1 and CTLA-4 inhibitors has been shown to broaden T-cell activation in multiple cancer types, offering a potential strategy to enhance immune responses in PROC. The phase II NRG-GY003 trial evaluated nivolumab (PD-1 inhibitor) alone or in combination with ipilimumab (CTLA-4 inhibitor) in ROC. The combination demonstrated a higher ORR of 16% compared to 6% with nivolumab alone and led to a median OS improvement (28.1 vs. 21.8 months), though statistical significance was not reached due to the small sample size. Notably, PFS nearly doubled (3.9 vs. 2.0 months), suggesting that dual checkpoint blockade may benefit a subset of patients [117].

To improve outcomes further, ongoing trials are evaluating additional checkpoint combinations. One emerging approach is the PD-1/LAG-3 blockade. LAG-3, an inhibitory receptor associated with T-cell exhaustion, is often upregulated after PD-1 therapy. Relatlimab, a LAG-3 inhibitor, has already been approved in combination with nivolumab for melanoma and is now under investigation in OC [146]. A bispecific antibody, tebotelimab, which targets both PD-1 and LAG-3 simultaneously, has demonstrated enhanced T-cell activation in early studies and may soon be tested in OC [147].

Other promising checkpoint targets include TIM-3 and TIGIT, which are expressed on TILs in OC [148]. TIM-3 blockade has been shown to reinvigorate T cells in preclinical models, and several clinical trials (e.g., NCT04137900) are now testing TIM-3 and TIGIT inhibitors in combination with PD-1/PD-L1 blockade in advanced gynecologic cancers [148,149]. These strategies aim to overcome resistance to single-agent checkpoint inhibitors and improve tumor control in immune-resistant OC.

#### 6.2.2. Combination Therapy: Checkpoint Inhibitors and Anti-Angiogenics

Anti-angiogenic therapy, particularly VEGF inhibition with bevacizumab, is a standard component of OC treatment and has been shown to modulate the TME by normalizing tumor vasculature and reducing immunosuppressive VEGF signaling [150]. Preclinical data suggest that VEGF blockade enhances T-cell infiltration, potentially improving checkpoint inhibitor efficacy [151]. Clinically, combinations of ICIs with anti-angiogenics have demonstrated improved activity, particularly in PROC [152].

A phase II trial of atezolizumab (PD-L1 inhibitor) with bevacizumab and chemotherapy (platinum and taxane) in platinum-sensitive OC relapse showed higher response rates than chemotherapy alone [153], though the IMagyn050 frontline trial in unselected patients did not show a significant PFS benefit [154]. In PROC, a non-randomized study at MD Anderson found that pembrolizumab + bevacizumab achieved an ORR of 40%, markedly higher than what is typically seen with either agent alone [155].

Building on these findings, a triplet regimen of pembrolizumab, bevacizumab, and low-dose metronomic cyclophosphamide (to enhance immunogenic cell death) was tested in a single-arm phase II trial and subsequent real-world settings. In a cohort of 40 heavily pretreated PROC patients, the combination achieved an ORR of 21–25%, with disease control in ~95% of patients [155]. A retrospective series of 55 patients reported even more impressive results, with an ORR of 42%, a median PFS of 7.8 months, and a median OS of 14 months – substantially better than historical chemotherapy outcomes in platinum-resistant disease. Nearly half of the patients achieved stable disease, leading to a clinical benefit rate of 91% [155]. These findings suggest that anti-angiogenic therapy combined with dual immune stimulation, via checkpoint inhibition and low-dose chemotherapy, can produce synergistic effects in PROC.

Ongoing randomized trials, such as IMagynist (NCT03740165), are now formally evaluating the ICI + bevacizumab + cyclophosphamide combination versus chemotherapy [156]. If confirmed, this regimen could represent a new therapeutic standard for PROC, particularly for patients without targetable mutations, expanding the role of immunotherapy in OC management.

In June 2024, Merck announced positive topline results from the phase 3 KEYNOTE-B96 trial, which evaluated pembrolizumab in combination with chemotherapy in patients with PROC. The study met its primary endpoint, demonstrating a statistically significant improvement in PFS compared to chemotherapy alone. According to the press release, “pembrolizumab plus chemotherapy significantly improved PFS compared to chemotherapy alone in both the PD-L1-positive (CPS ≥ 1) population and the all-comer population” [157]. Specifically, in the PD-L1-positive group, the median PFS was 5.6 months vs. 4.0 months with chemotherapy alone, and in the overall population, 5.3 months vs. 3.8 months [158].

#### 6.2.3. Combination Therapy: Checkpoint Inhibitors and PARP Inhibitors

PARPi target tumor DNA repair deficiencies, leading to accumulated DNA damage, increased neoantigen release, and heightened T-cell recruitment. Preclinical studies suggest that PARPi-induced DNA damage activates the STING pathway and type I interferon signaling, potentially enhancing tumor immunogenicity and responsiveness to ICIs. However, clinical results from PARPi-ICI combinations have been mixed, with efficacy appearing most pronounced in *BRCA*-mutated and HRD OCs [159].

The MEDIOLA trial, which evaluated durvalumab (PD-L1 inhibitor) plus olaparib in *BRCA*-mutated platinum-sensitive OC, demonstrated an ORR of ~72%, with prolonged disease control, highlighting the synergy between PARP inhibition and immune activation in tumors with DNA repair defects [119]. In the platinum-resistant setting, the TOPACIO/KEYNOTE-162 trial assessed niraparib (PARPi) plus pembrolizumab (PD-1 inhibitor) and reported a modest ORR of ~18%, which increased to 28% in *BRCA*-mutated patients, reinforcing the role of genomic biomarkers in patient selection [160].

Despite these encouraging signals, not all trials have yielded positive results. The MOONSTONE/GOG-3032 phase III trial of niraparib plus dostarlimab (PD-1 inhibitor) in PROC was terminated early after an interim analysis suggested it was unlikely to outperform chemotherapy. This underscores that PARPi-ICI combinations are most effective in tumors with inherent DNA repair defects, where the induction of neoantigens and immune activation is more pronounced [161].

Ongoing large-scale trials continue to explore multimodal strategies. One key study is DUO-O, which evaluated the addition of durvalumab to chemotherapy and bevacizumab, followed by maintenance with durvalumab and olaparib. In the homologous recombination-deficient (HRD), *BRCA* wild-type population, this arm showed a significant median PFS of 45.1 months versus 23.3 months in the control arm (HR 0.46) [162]. However, in the intention-to-treat population, the benefit was more modest (25.1 vs. 19.3 months; HR 0.61), and there was no statistically significant OS improvement (HR 0.95; 95% CI 0.76–1.20). Toxicity remains a concern, with grade ≥3 neutropenia in 51% of patients and treatment discontinuation rates of 35% in the triplet arm compared to 22% in the control arm [163]. Similarly, the ATHENA trial is investigating a comparable regimen in both frontline and recurrent settings [164]. Taken together, these findings suggest that although PFS gains are promising, the lack of OS benefit and overlapping hematologic and hepatic toxicity warrant careful patient selection and monitoring.

While PARPi-ICI combinations have not yet redefined the standard of care for PROC, they remain a promising strategy for HRD-positive tumors, potentially offering dual therapeutic benefit through PARP-induced tumor cell death and enhanced immune recognition.

#### 6.2.4. Other Novel Checkpoint Targets

Beyond PD-1/PD-L1 and CTLA-4, emerging checkpoint targets are being explored to overcome the immunosuppressive TME in PROC. These newer approaches aim to enhance T-cell activation, relieve macrophage and DC inhibition, and promote anti-tumor immunity.

B7-H4 (VTCN1), a B7 family ligand highly expressed in OC, inhibits T-cell function. The B7-H4-targeting antibody–drug conjugate XB002 (ACI-Iconic) is currently in early clinical trials, with potential dual effects of direct tumor killing and immune modulation [165]. Another target, IDO1, suppresses immune responses by depleting tryptophan, an essential amino acid for T-cell function. While epacadostat (IDO1 inhibitor) failed in melanoma trials, combinations such as epacadostat + pembrolizumab were investigated in OC but did not demonstrate clear clinical benefit [166]. However, new, more potent IDO inhibitors and broader indoleamine pathway blockers are in development.

Co-stimulatory checkpoint agonists, such as CD27 and OX40, are also being tested to enhance T-cell activation alongside PD-1 blockade. The CD27 agonist varlilumab, in combination with nivolumab, showed modest activity (12.5% ORR) in OC with an acceptable safety profile [167]. Macrophage-targeted checkpoint inhibitors, including SIRPα inhibitors (which block the tumor CD47 “don’t eat me” signal), are being evaluated in OC [143]. Agents such as tiragolumab (anti-TIGIT) and magrolimab (anti-CD47) aim to relieve immune suppression by myeloid cells and are being combined with ICIs or chemotherapy in clinical trials [168,169].

To broaden immune activation, novel bispecific checkpoint antibodies are being developed. Regeneron’s CD28 co-stimulatory bispecific antibody (REGN5668) is currently under investigation with MUC16 × CD3 bispecific therapy, designed to enhance T-cell activation within the TME [170].

As checkpoint blockade therapy evolves in OC, single-agent PD-1 inhibitors are giving way to multifaceted immunotherapy regimens incorporating novel checkpoint targets. The goal is to convert immune-cold PROC tumors into immune-responsive tumors, thereby increasing the proportion of patients achieving durable responses. Ongoing trials will determine which checkpoint combinations offer the most effective and clinically viable strategies for PROC treatment.

### 6.3. Cancer Vaccines

Cancer vaccines aim to stimulate the immune system to recognize and attack tumor-specific antigens, offering a promising strategy for OC, which expresses well-defined targets such as CA-125 (MUC16), NY-ESO-1, and WT1. Recent advances, particularly in mRNA vaccine technology, have renewed interest in vaccine-based immunotherapy for PROC. Current approaches include DC vaccines, peptide or protein subunit vaccines, personalized neoantigen vaccines, and viral vector or nucleic acid-based (DNA/RNA) vaccines.

#### 6.3.1. mRNA-Based Vaccines

The success of mRNA vaccines in infectious diseases has accelerated their application in oncology, offering a platform to encode tumor antigens or neoantigens and induce robust T-cell and antibody responses. Several biotech companies are advancing mRNA vaccine trials for OC, with a focus on personalized and shared antigen-targeting approaches.

BioNTech has integrated an mRNA vaccine (CARVac) into its BNT211 CAR-T regimen and is also developing a separate individualized mRNA vaccine program [171]. Their IVAC mutanome approach, which was proven in melanoma, is now being tested in OC patients, identifying tumor-specific mutations and encoding them in an mRNA cocktail [172]. Moderna is conducting a phase I trial (MODI-1) evaluating a personalized mRNA vaccine in solid tumors, including OC. While the results are pending, preclinical data suggest that mRNA vaccines can induce cytotoxic T-cell responses against OC cells [173].

Researchers at the University of Oxford are developing OvarianVax, a program aiming to create both a prophylactic vaccine for high-risk individuals and therapeutic vaccines for OC patients [174]. One candidate, an mRNA vaccine encoding fragments of the CA-125 (MUC16) neoantigen, has demonstrated immune activation in preclinical OC models [124].

The flexibility of mRNA technology allows for the simultaneous targeting of multiple antigens. For example, BioNTech’s BNT122 (autogene cevumeran), a personalized mRNA vaccine used in melanoma, could theoretically be adapted to cover dozens of patient-specific neoantigens in OC [175]. However, one challenge is the lower mutational burden of OC compared to melanoma, limiting the availability of highly immunogenic neoantigens. Nonetheless, even shared mutations, such as TP53 alterations (present in >90% of HGSC), could be leveraged as vaccine targets [176].

Several early-phase clinical trials of mRNA vaccines in OC are currently ongoing, with results expected in the next 1–2 years. These studies will provide critical insights into immunogenicity, safety, and potential clinical efficacy, shaping the future role of mRNA vaccine technology in OC immunotherapy.

#### 6.3.2. Dendritic Cell Vaccines

DC vaccines leverage the potent antigen-presenting capability of DCs to prime naïve T cells and elicit tumor-specific immune responses. These vaccines are generated by isolating patient monocytes, differentiating them into DCs, loading them with tumor antigens, and re-infusing them to stimulate anti-tumor immunity.

A leading example in OC is DCVAC/OvCa, developed by SOTIO, an autologous DC vaccine loaded with killed OC cells to present a broad range of tumor antigens. In a phase II trial for first-relapse platinum-sensitive OC, DCVAC plus chemotherapy did not extend PFS but significantly improved OS. The DCVAC-treated group had a median OS of 35.5 months, compared to 22.1 months in the chemotherapy-only group, representing a 13.4-month survival benefit and a 62% reduction in the risk of death. While PFS remained similar between groups, the ORR was higher with DCVAC (88% vs. 63%), suggesting delayed but durable immune-mediated tumor control. This “survival despite progression” phenomenon, observed in some vaccine and ICI trials, indicates that DCVAC may induce long-lived anti-tumor immunity that slows tumor regrowth post-progression [177].

Building on the results of the SOV02 phase II trial, which showed improved OS with DCVAC/OvCa in first-relapse platinum-sensitive OC [177], SOTIO continues to investigate DC-based immunotherapy, and multiple strategies are being explored to enhance DC vaccine efficacy in OC. One such approach involves the use of DCs pulsed with oxidized autologous whole-tumor lysates (OCDC). In a clinical study, this vaccine induced tumor-specific T-cell responses and was associated with prolonged survival in patients with ROC [178]. Another promising direction involves DC vaccines generated from patient-derived DCs fused with allogeneic tumor cells, which aim to present a broader repertoire of tumor antigens. In a phase I/II trial at the University of Pennsylvania, this fusion vaccine—administered with or without cyclophosphamide—achieved a 3-year OS rate of 90% in OC patients in remission [179].

In PROC, DC vaccines are now being combined with ICIs to enhance anti-tumor responses. A recent Japanese study combined a WT1 peptide-pulsed DC vaccine with the PD-1 inhibitor nivolumab in patients with WT1-positive OC in second or third remission. The combination was well tolerated and induced WT1-specific T-cell responses, with a one-year PFS rate of 70%, suggesting a synergistic immunologic effect [180]. These findings support further investigation of DC vaccines in both monotherapy and combination strategies, particularly in settings of minimal residual disease or recurrence.

Another promising avenue is the use of DC vaccines as maintenance therapy following tumor debulking or chemotherapy, aiming to eliminate minimal residual disease and prevent recurrence [181]. Several ongoing trials are evaluating this strategy, seeking to establish DC vaccines as a viable immunotherapeutic option for PROC and ROC.

#### 6.3.3. Peptide and Protein Vaccines

Peptide and protein-based vaccines stimulate anti-tumor immunity by presenting specific TAAs to the immune system, often with an adjuvant to enhance response. While early trials in OC have shown immunogenicity, demonstrating objective tumor regression remains a challenge, leading to efforts to refine targets and enhance vaccine efficacy through combination strategies.

One of the earliest OC vaccine candidates was abagovomab (OVA-301), an anti-idiotype antibody that mimics CA-125. However, the phase III MIMOSA trial evaluating abagovomab as a maintenance vaccine failed to improve clinical outcomes [182]. More recent vaccine development has focused on more immunogenic targets, including NY-ESO-1, FRα, and survivin.

NY-ESO-1, a cancer–testis antigen expressed in ~40% of ovarian tumors, has been targeted with a long peptide vaccine plus Toll-like receptor (TLR) agonist adjuvant, which induced T-cell responses in a subset of patients, with some cases of delayed progression in early trials [183]. Similarly, a peptide vaccine targeting FRα (E39, folate-binding protein peptide vaccine) demonstrated immunogenicity and safety in a phase I/II study, though larger trials are required to determine efficacy [184]. Another investigational vaccine, DPX-Survivac, which targets survivin, has also shown immune activation in OC patients (phase II, NCT02785250) [185].

To enhance efficacy, peptide vaccines are frequently combined with immune modulators. Poly-ICLC (a TLR3 agonist) is used as an adjuvant to promote Th1-skewed immune responses [186], while low-dose cyclophosphamide has been incorporated to reduce Tregs and enhance vaccine-induced immunity [187]. Companies such as CureVac and BioNTech are also developing RNA-based or DC-loaded peptide vaccines targeting OC antigens [188].

A novel approach by CureLab Oncology utilizes a DNA plasmid vaccine encoding p62/SQSTM1, a protein involved in tumor metabolism and immunity (branded Elenagen). In a first-in-human study in Eastern Europe, the p62 DNA vaccine combined with chemotherapy reportedly delayed disease progression in PROC, suggesting potential for chemosensitization [189]. CureLab is now planning further trials in PROC patients in the U.S. [190].

Although peptide and protein vaccines alone have not consistently induced objective tumor shrinkage, they generate specific immune responses that may extend survival or enhance combination immunotherapy strategies in OC. Ongoing trials continue to explore their potential as part of multi-modal treatment approaches.

#### 6.3.4. Neoantigen Vaccines

Neoantigen vaccines are a highly personalized immunotherapy approach designed to stimulate T-cell responses against tumor-specific mutations. These vaccines are developed by sequencing a patient’s tumor, identifying immunogenic neoantigens, and encoding them into peptides, RNA, or viral vectors for vaccination. This strategy has demonstrated potent immune responses in melanoma and glioblastoma and is now being explored in OC [191].

A proof-of-concept study in OC utilized DC vaccines pulsed with patient-specific neoantigen peptides, demonstrating that these vaccines can elicit tumor-infiltrating T cells and stabilize disease. In one pilot trial, ROC patients were vaccinated with up to 10 neoantigen peptides plus poly-ICLC adjuvant. Several patients developed neoantigen-specific T-cell responses, and one patient experienced disease stability for over a year, suggesting potential clinical benefit [178].

Given OC’s relatively low mutation burden, neoantigen vaccines may be most effective in tumors with DNA repair deficiencies (e.g., *BRCA*-mutated or HRD tumors), which have a higher neoantigen load [192]. Additionally, combining neoantigen vaccines with checkpoint inhibitors may enhance T-cell expansion and persistence, overcoming immunosuppressive mechanisms in the TME [193].

Several biotech companies, including Genocea [194], ISA Pharmaceuticals [195], and Frame Therapeutics [196], are incorporating OC cohorts into their personalized vaccine trials, investigating their efficacy in solid tumors with intermediate mutation rates. One challenge in OC is identifying sufficient high-quality neoantigens, as many tumor mutations are passenger mutations that are poorly expressed or weakly presented on HLA molecules [176].

To overcome this, researchers are also investigating “shared” neoantigen vaccines that target recurrent mutations found across multiple patients, such as common *TP53* hotspot mutations (seen in >90% of HGSC) [197] or *KRAS* mutations in clear-cell ovarian cancer [198]. These approaches aim to broaden neoantigen vaccine applicability and increase accessibility for OC patients. Ongoing trials will determine whether neoantigen-based strategies can achieve durable immune responses and clinical benefit in PROC.

#### 6.3.5. Other Vaccine Modalities

Several innovative vaccine strategies are being explored to enhance immune responses in OC, beyond peptide-, DC-, and neoantigen-based approaches. Viral vector vaccines, such as modified poxviruses (e.g., PANVAC, which delivers CEA and MUC1 antigens along with co-stimulatory molecules), have been tested in OC, inducing immune responses but demonstrating limited efficacy as monotherapy [199]. Similarly, oncolytic virus vaccines, which will be discussed in Section 6.5, act as an in situ vaccination strategy by infecting tumors and releasing neoantigens in an inflammatory context, potentially enhancing tumor-specific immunity.

A recent approach involves whole-tumor-cell vaccines, which use a patient’s own inactivated tumor cells to provide a broad array of tumor antigens. One such vaccine, Innocell, utilizes a hypoxic photochemical process to inactivate tumor cells, preserving antigenic integrity. In 2022, the FDA approved a phase I trial of this personalized whole-cell vaccine for stage III/IV OC, representing a novel autologous vaccination strategy [200].

Another emerging concept is combination vaccines that simultaneously target tumor cells and immunosuppressive Tregs. For example, a vaccine targeting both folate receptor on Tregs and an ovarian tumor antigen could simultaneously activate effector T cells while reducing Treg-mediated immune suppression, potentially improving overall vaccine efficacy [201].

OC vaccines are experiencing a resurgence due to advancements in personalized neoantigen platforms, DC vaccines, and combination immunotherapy approaches. While single-antigen vaccines have historically shown limited clinical benefit, modern multi-targeted and immune-enhancing strategies hold greater promise. A key area of investigation is combining vaccines with other immunotherapies, for instance, administering a vaccine before a checkpoint inhibitor to expand the tumor-specific T-cell pool that can be subsequently activated by PD-1 blockade.

As ongoing trials mature, the field will determine whether vaccines can meaningfully extend remission or prevent recurrence in PROC. While vaccines alone are unlikely to be curative in advanced disease, they may serve as maintenance therapy to prevent relapse after chemotherapy or as adjuvants to cellular immunotherapies, such as in the CAR-T plus CARVac strategy. The coming years will reveal whether mRNA and neoantigen vaccines can replicate the strong immune responses observed in melanoma and establish a meaningful role in OC treatment.

### 6.4. Bispecific T-Cell Engagers (TCEs) and Dual-Target Antibodies

While cell-based therapies, checkpoint inhibitors, and cancer vaccines have dominated immunotherapy development, additional innovative immune-based approaches are being explored for PROC. One such strategy involves bispecific TCEs and dual-target antibodies, which aim to enhance tumor-directed immune responses by bridging T cells directly to cancer cells.

Bispecific TCEs are engineered antibodies that bind a tumor antigen with one arm and CD3 on T cells with the other, redirecting T cells to attack cancer cells independent of their natural T-cell receptor specificity. In OC, MUC16 (CA-125) has emerged as a key TCE target. Regeneron’s ubamatamab (REGN4018), an MUC16 × CD3 bispecific antibody, has shown promising early clinical activity. In a phase I dose-escalation study (ESMO 2022), partial responses were observed in a subset of PROC patients, particularly those with high MUC16 expression. The ORR was modest (~6–12%), but increased to 21% in patients without visceral metastases and 31% in those with high MUC16 expression, suggesting dose-dependent activity. The therapy was generally well tolerated, with manageable CRS in some patients—an expected on-target effect of TCEs [202].

To enhance T-cell responses, Regeneron is now testing ubamatamab in combination with cemiplimab (PD-1 inhibitor), hypothesizing that checkpoint blockade may improve T-cell persistence and tumor killing. In addition, they are developing a CD28 co-stimulatory bispecific (REGN5668), which binds MUC16 and CD28 to provide a secondary activation signal to T cells at the tumor site. Preclinical studies suggest that combining a CD3-engaging TCE with a CD28 bispecific greatly enhances T-cell proliferation and tumor eradication, without systemic T-cell overactivation, as co-stimulation is restricted to MUC16-positive tumor cells [202].

Beyond MUC16, other bispecifics targeting FRα and CD3 are in development, following a similar approach. For example, MacroGenics is investigating a B7-H3 × CD3 bispecific (derivative of enoblituzumab), as B7-H3 is broadly expressed in OC [203]. Additionally, trispecific antibodies, which target a tumor antigen, CD3, and an NK-cell receptor, are being explored preclinically to engage multiple immune cell populations simultaneously [204], further expanding the bispecific therapeutic landscape.

While TCEs and dual-target antibodies remain early in development for OC, their ability to redirect immune cells toward tumors with specificity offers an exciting avenue for immune-based treatment strategies in PROC. Ongoing clinical trials will clarify their efficacy, safety, and optimal combination strategies, potentially positioning them alongside mainstream immunotherapies in the future.

### 6.5. Oncolytic Viruses

Oncolytic virotherapy utilizes genetically engineered viruses that selectively infect and lyse tumor cells while simultaneously stimulating anti-tumor immunity. Given that OC often disseminates within the peritoneal cavity, direct IP administration of oncolytic viruses provides a promising approach to target metastatic tumor deposits and enhance immune infiltration.

A major advancement in this field is Olvimulogene nanivacirepvec (Olvi-Vec), a modified vaccinia virus (GL-ONC1 strain) evaluated in the phase II VIRO-15 trial for PROC or ROC. Olvi-Vec is administered intraperitoneally, where it infects and lyses tumor cells, releasing neoantigens and pro-inflammatory signals. A unique aspect of VIRO-15 was its “prime-and-adopt” strategy, where patients received Olvi-Vec as a priming agent, followed 7–10 days later by chemotherapy (platinum doublet + bevacizumab) to exploit the virus-induced immune activation [205].

The 2023 trial results demonstrated remarkable efficacy in a heavily pretreated PROC population: among 24 evaluable patients, 54% (13 patients) achieved an objective response (RECIST criteria), including several complete responses following chemotherapy. The median PFS was 11.0 months, significantly longer than the ~3–4 months typically observed with chemotherapy alone in similar patients. Correlative studies showed that Olvi-Vec promoted immune cell infiltration and inflammatory cytokine release in the TME, potentially reversing platinum resistance by sensitizing tumors to chemotherapy. The safety profile was favorable, with manageable fever and abdominal discomfort related to viral infection but no unexpected severe toxicities. These findings led to the initiation of the phase III OVERVEIL trial (NCT05281471) to confirm the benefit of Olvi-Vec immunochemotherapy vs. chemotherapy alone [206].

Other oncolytic viruses are also in clinical development for OC. Pelareorep (Reolysin), an oncolytic reovirus, has been tested in combination with paclitaxel [207]. ONCOS-102, an adenovirus expressing GM-CSF, is being investigated with durvalumab for peritoneal malignancies [208]. HSV-1-based oncolytic viruses, such as talimogene laherparepvec (T-VEC) [209] and oHSV-2 (ONCR-177), are being explored for direct injection into ovarian tumors via laparoscopy [210].

Beyond direct tumor lysis, oncolytic viruses act as in situ cancer vaccines, releasing tumor antigens within an inflammatory context, leading to enhanced immune cell recruitment. This property is particularly valuable for immunologically “cold” tumors like OC, which otherwise exhibit poor spontaneous T-cell infiltration [209]. The synergy between Olvi-Vec and chemotherapy/anti-VEGF agents represents one of the most promising immunomodulatory strategies for overcoming platinum resistance, with potential to reshape therapeutic paradigms in PROC.

### 6.6. Cytokine and Immunomodulatory Therapies

Cytokines and immune modulators are key regulators of anti-tumor immune responses and are being actively investigated for PROC. While high-dose IL-2 was among the first immunotherapies for cancer, its toxicity limited clinical use, prompting the development of engineered cytokines with improved selectivity and reduced adverse effects.

Interleukin-2/15 Superkines are being designed to enhance effector T-cell and NK-cell activity while avoiding stimulation of Tregs. Nemvaleukin alfa (ALKS 4230), a fusion of IL-2 and IL-2 receptor, preferentially activates IL-2Rβγ on cytotoxic immune cells while sparing IL-2Rα-mediated Treg expansion. In a phase I trial, nemvaleukin alone or with pembrolizumab showed a 28.6% response rate in one PROC cohort, leading to the phase III ARTISTRY-7 trial, which is now fully accrued and will compare nemvaleukin + pembrolizumab vs. chemotherapy in PROC. If successful, this could establish cytokine-based immunotherapy as a viable strategy [211]. Similarly, IL-15 superagonists (such as IL-15N72D/IL-15Rα, also known as N-803) are being tested to support memory T- and NK-cell expansion without Treg activation. An IL-15 superagonist combined with a cancer vaccine in OC has shown immune activation [212], though clinical efficacy data are still pending.

Another cytokine-based strategy involves interferon-alpha (IFN-α), which has been used intraperitoneally in OC, showing modest clinical benefit but high toxicity. To improve safety, IFN-α gene therapy and IFNAR agonists are being tested to stimulate immune responses within the TME [213].

Targeting TGF-β, a key immunosuppressive cytokine in OC, is another promising approach. M7824 (bintrafusp alfa), a bifunctional antibody that blocks PD-L1 and sequesters TGF-β, showed some disease control in OC, though response rates were low [214]. However, localized inhibition of TGF-β within the peritoneal cavity remains an area of active research.

Other strategies focus on stimulating innate immune pathways. The STING (stimulator of interferon genes) pathway plays a crucial role in linking tumor DNA sensing to immune activation. STING agonists such as ADU-S100 and MK-1454, when injected into tumors, can induce type I interferons and promote T-cell infiltration. Ongoing trials are evaluating whether IP STING agonists can convert immunologically “cold” ovarian tumors into immune-responsive tumors [215]. Similarly, TLR agonists have been tested in OC, though early trials, such as TLR8 agonist motolimod plus pegylated doxorubicin, did not improve outcomes—possibly due to insufficient immune infiltration. However, TLR agonists in combination with PD-1 blockade are being revisited to enhance efficacy [216].

The adenosine pathway, which contributes to immunosuppression in the ovarian TME, is another target for immunotherapy. Inhibitors of CD73 (an enzyme that generates immunosuppressive adenosine) or A2A adenosine receptor antagonists aim to relieve T-cell inhibition. CPI-444 (A2A receptor antagonist) plus atezolizumab showed prolonged stable disease in some OC patients, supporting further investigation in combination regimens to address multiple mechanisms of immune resistance [217].

These cytokine and immunomodulatory approaches highlight the potential of targeting the TME to overcome immune evasion in PROC. While many of these agents are in early-phase trials, their integration into combination immunotherapy regimens may provide new treatment avenues for immune-refractory OC.

### 6.7. Engineered T-Cell Receptor (TCR) Therapies

Engineered TCR therapies expand the scope of adoptive T-cell therapy by allowing T cells to recognize intracellular tumor antigens presented on major histocompatibility complex (MHC) molecules. Unlike CAR-T cells, which are restricted to surface antigens, TCR-T cells can target intracellular oncogenic proteins [218], making them a potential therapeutic option for PROC.

One of the earliest OC applications of TCR therapy targeted NY-ESO-1, a cancer–testis antigen expressed in approximately 40% of ovarian tumors. In a small clinical study, NY-ESO-1-specific TCR T-cell therapy was administered to OC patients, yielding a partial response in one patient. However, responses were not durable, and many tumors exhibited low or heterogeneous expression of NY-ESO-1, limiting efficacy [218].

To improve TCR targeting strategies, newer TCR therapies directed against mutated intracellular proteins such as p53 [219] and KRAS [220]—both frequently altered in OC—are now in preclinical development. These mutant-specific TCRs could provide a more selective approach, targeting tumor-driving mutations while sparing normal tissues.

Despite their potential, TCR therapies face key challenges in PROC. The requirement for HLA matching limits broad applicability, and downregulation of MHC expression, a common immune evasion mechanism in OC, may reduce antigen presentation and TCR-T-cell efficacy [221]. To counteract this, combination approaches are being explored, such as TCR therapy with MHC upregulators like interferons, epigenetic modulators, or checkpoint inhibitors to enhance tumor antigen presentation [222].

While still early in development, TCR therapies offer a precision-based immunotherapy approach for OC subtypes expressing targetable intracellular antigens. Future trials will determine whether optimized TCR designs and combinatorial strategies can overcome MHC limitations and improve clinical efficacy in immune-resistant OC.

### 6.8. Miscellaneous Approaches

Several emerging immunotherapeutic strategies are being explored for PROC, aiming to enhance T-cell activation, reprogram the TME, and optimize immune responses.

One promising approach targets folate receptor beta (FRβ) on TAMs to selectively deplete immunosuppressive macrophages and promote a more immune-permissive environment. Monoclonal antibodies such as m909 have shown preclinical efficacy in targeting FRβ-expressing TAMs [223]. MIW815 (also known as ADU-S100), a STING agonist, is in early development to activate innate immunity through type I interferon production [224]. Another innovative strategy utilizes vaccinia-based B7-1/ICAM-1/Leukine (Triad of Co-stimulatory Molecules [TRICOM]) vectors, designed to enhance co-stimulatory molecule expression within tumors, effectively making tumor cells more efficient at activating T cells [225].

Checkpoint agonists, rather than inhibitors, are also being tested to activate key immune cells in cancer immunotherapy. CD40 agonists, which stimulate DCs and enhance antigen presentation, have shown promise in preclinical OC models. One example is SL-172154, a bispecific molecule combining CD47 blockade with CD40 activation, currently being evaluated in a phase I trial (NCT04406623) for patients with PROC [226].

Additionally, growing evidence suggests that the gut microbiome influences immunotherapy responses in OC. Certain gut bacteria have been linked to improved responses to ICIs, leading to early discussions on microbiome modulation as an adjunct to immunotherapy. Strategies such as probiotics or fecal microbiota transplantation (FMT) are being explored to enhance systemic immune responses if the initial findings hold up in clinical trials [227].

Collectively, these approaches illustrate an evolving multipronged strategy against PROC, encompassing T-cell engagement via bispecific antibodies, innate immune activation through oncolytic viruses and cytokines, and TME reprogramming. While many of these novel immunotherapies remain experimental, several, including oncolytic virus priming and bispecific TCEs, have already shown clinically meaningful tumor responses. The next major challenge will be integrating these diverse strategies into combination regimens that maximize synergy. For example, an oncolytic virus could recruit T cells, a bispecific T-cell engager could direct them to tumor targets, and ICIs could sustain their activation.

With an expanding arsenal of immunotherapies, post-2021 developments offer new hope that even highly refractory ovarian cancer may be rendered more immunologically responsive, bringing immunotherapy closer to clinical relevance in PROC.

## 7. Overcoming Challenges and Future Directions

Despite significant advancements in immunotherapy for PROC, major obstacles continue to impede durable responses. Tumor heterogeneity, immune suppression within the TME, and resistance mechanisms remain formidable barriers that blunt even the most potent immunotherapies. Addressing these challenges will require a strategic shift from simply adding immunotherapy to existing treatments to reengineering the immune landscape itself. This section examines the key limitations of current approaches, explores next-generation strategies, and outlines emerging combination paradigms that could fundamentally reshape the treatment landscape for PROC.

### 7.1. Challenge 1: The Immunosuppressive TME

The TME of PROC is highly immunosuppressive, often rendering checkpoint inhibitors and other immunotherapies ineffective. Ovarian tumors typically exhibit low effector T-cell infiltration (“immune-cold” phenotype) while being dominated by TAMs, MDSCs, and Tregs, which collectively inhibit immune responses [143]. These suppressive cells secrete IL-10, TGF-β, VEGF, and upregulate PD-L1, further dampening T-cell function [228]. Additionally, peritoneal ascites fluid, commonly present in OC, is rich in immunosuppressive cytokines and may dilute infused immune cells, further weakening therapeutic efficacy [228].

Overcoming these barriers requires strategies to reprogram or deplete suppressive immune cells. CSF1R inhibitors can target TAMs [144], while CXCR2 inhibitors may block MDSC recruitment [229]. Another strategy involves TME-modulating immune adjuvants, such as STING agonists or oncolytic viruses, to inflame tumors and enhance immune cell infiltration [215]. A promising example is the Olvi-Vec trial, which demonstrated how an oncolytic virus can convert an immune-cold tumor into a hot one, followed by chemotherapy to sustain the immune response [205,206]. Future trials may explore adding checkpoint inhibitors (ICIs) to further enhance anti-tumor activity.

Local IP therapies are another avenue to maximize immune activation while limiting systemic toxicity. Trials are evaluating IP IL-12 gene therapy (GEN-1 nanoparticles) alongside chemotherapy to activate local immunity. Early findings suggest that GEN-1 may improve immune cell infiltration, with preliminary data showing a trend toward better PFS in newly diagnosed patients [230].

In the future, “TME conditioning” regimens may be employed before immunotherapy to reduce physical and biochemical barriers to immune cell infiltration. For instance, short courses of anti-VEGF therapy, low-dose radiotherapy, or epigenetic modulators could increase MHC expression on tumor cells, enhance chemokine secretion, and improve immune cell trafficking to the tumor site [231]. The concept of radiation as an immune adjuvant (“abscopal effect”), where local irradiation enhances systemic anti-tumor immunity, is currently being tested in pilot studies for OC [232].

Addressing the immunosuppressive TME will be essential to unlock the full potential of immunotherapy in PROC, making tumors more permissive to T-cell infiltration and activation, and ultimately improving clinical outcomes.

### 7.2. Challenge 2: Tumor Heterogeneity and Antigen Escape

Tumor heterogeneity and antigen escape present significant obstacles to immunotherapy in PROC. Ovarian tumors exhibit both molecular and spatial heterogeneity, meaning different metastatic lesions and even regions within the same tumor can have distinct antigen expression profiles and immune markers [106]. This variability complicates targeted immunotherapies such as CAR-T cells and bispecific antibodies, as antigen-negative clones within a tumor can evade immune recognition, leading to resistance and relapse [120].

A well-documented mechanism of immunotherapy resistance is antigen downregulation or loss, which has been observed in CAR-T and TIL therapy for solid tumors. For example, a patient treated with MUC16-targeted CAR-T cells might initially respond but relapse with MUC16-negative tumors, rendering the therapy ineffective [125]. To mitigate this risk, next-generation approaches are focusing on multi-antigen targeting to enhance coverage and reduce immune escape.

One strategy involves engineering dual or tandem CAR-T cells, where two distinct CARs (e.g., mesothelin and FRα) are expressed on the same T cell, ensuring that tumor cells with heterogeneous antigen expression remain vulnerable to attack [120]. Similarly, bispecific TCEs targeting two different tumor antigens or even trispecific engagers are under development to broaden immune engagement [202].

Personalized approaches to antigen selection may further improve treatment efficacy. By profiling individual tumor antigen expression, customized CAR-T or TCR therapies can be designed to target multiple highly expressed antigens in a given patient [233]. Advances in CRISPR gene editing now allow for the insertion of multiple CARs or TCRs into a single T cell (TanCAR-T), a strategy that may feature in future clinical trials to enhance antigen coverage [234].

On the cancer vaccine front, neoantigen-based vaccines inherently address tumor heterogeneity by tailoring treatment to each patient’s tumor mutanome. However, tumors can still evade immune detection through neoantigen loss or downregulation of HLA molecules, which limits T-cell recognition [198].

To combat dynamic antigenic changes, regular tumor biopsy and molecular monitoring (e.g., circulating tumor DNA, ctDNA, sequencing) during immunotherapy are likely to become standard practice. This adaptive therapy approach would enable real-time adjustments to immunotherapy strategies, allowing for the early detection of antigen escape and selection of alternative immune targets or combination treatments [235]. As multi-antigen targeting, personalized immunotherapies, and real-time molecular monitoring become more integrated into clinical practice, immune escape in OC may be better controlled, improving the durability of responses to immunotherapy.

### 7.3. Challenge 3: Immune Checkpoint and Exhaustion Pathways

Immune checkpoint upregulation and T-cell exhaustion present significant barriers to effective immunotherapy in PROC. While PD-1 and CTLA-4 blockade has revolutionized treatment in some malignancies [108,109,236], ovarian tumors frequently deploy multiple redundant checkpoint pathways, including TIM-3, LAG-3, and TIGIT, to evade immune attack [148,149,169]. When one inhibitory pathway is blocked, tumors often compensate by activating alternative checkpoints, limiting the efficacy of single-agent ICIs. The future of checkpoint blockade in OC will likely require simultaneous or sequential inhibition of multiple checkpoints, though excessive blockade risks severe immune-related toxicity.

To circumvent systemic toxicity, novel approaches are embedding checkpoint resistance directly into cell therapies. CRISPR gene editing has enabled PD-1-knockout T cells, preventing immune suppression by PD-L1-rich TMEs. A first-in-human study in melanoma (2022) tested CRISPR-edited PD-1-deficient TILs [237], and similar strategies could be applied to TIL or CAR-T therapies in OC. Additionally, “armored CAR-T cells” are being designed to secrete checkpoint-blocking single-chain variable fragments (scFvs) – essentially mini-antibodies that neutralize PD-1 within the tumor [238]. Other armored CAR-T strategies include built-in PD-1 blockade or dominant-negative PD-1 receptors, making engineered T cells resistant to PD-L1 inhibition without requiring systemic ICI administration [239].

Beyond classical checkpoint pathways, targeting metabolic immune checkpoints is emerging as a potential strategy. Ovarian tumors exploit metabolic suppression mechanisms, such as depleting tryptophan and producing immunosuppressive kynurenine via the IDO pathway, which paralyzes T-cell responses. Next-generation therapies may include IDO inhibitor gene-modified T cells or the controlled release of arginine to counteract immunosuppressive myeloid cells expressing arginase [240].

Given the complexity of checkpoint-driven immune evasion, a multi-modal approach will likely be necessary to enhance immunotherapy efficacy in OC. Future strategies may integrate cellular therapies or bispecific TCEs to deliver direct cytotoxic immune responses, while simultaneously incorporating localized checkpoint blockade engineered within the therapy itself to prevent T-cell exhaustion. Additionally, modulating the TME through agents such as TAM reprogrammers or metabolic inhibitors could help counteract immunosuppressive mechanisms, ultimately improving the durability and effectiveness of immune-based therapies.

By integrating genetic modifications, localized checkpoint inhibition, and TME reprogramming, next-generation immunotherapies could overcome immune exhaustion and enhance durable responses in OC while minimizing systemic toxicity.

### 7.4. Challenge 4: Lack of Predictive Biomarkers for Immunotherapy Response

Unlike in other cancers, no universally accepted biomarker currently predicts which OC patients will respond to immunotherapy. Standard markers such as PD-L1 expression and tumor mutation burden (TMB), which guide checkpoint inhibitor (ICI) use in malignancies like lung cancer and melanoma, have shown limited predictive value in OC [176]. Many PD-L1-positive ovarian tumors still fail to respond to PD-1 inhibitors, and TMB is generally low in OC, except in the rare microsatellite instability-high (MSI-H) subset, where ICIs have demonstrated some efficacy [241]. Although *BRCA*-mutated and HRD tumors generate more neoantigens, making them theoretically more immunogenic, clinical trials have shown no significant correlation between *BRCA* status and ICI response [242], though *BRCA*-mutant tumors do benefit from PARPi [243].

The need for better biomarkers to stratify patients and optimize immunotherapy selection is evident. Emerging candidates include immune gene signatures and measures of T-cell receptor clonality. Recent analyses suggest that an “inflamed” gene signature, characterized by high expression of CXCL9, CD8A, and interferon-gamma-related genes, correlates with better ICI and vaccine responses [244]. Additionally, the presence of tertiary lymphoid structures (TLS) within tumors, which serve as sites for local T-cell activation, has been associated with improved immune responses in other cancers and may prove relevant in OC [245].

Another promising approach is assessing tumor-reactive T cells in either peripheral blood or tumor biopsies. If a patient’s T cells already recognize a known antigen, therapies such as vaccines targeting that antigen or ICIs to unleash pre-existing T-cell responses could be more effective [246]. Some trials are incorporating T-cell clonotype tracking, where the expansion of dominant T-cell clones post-therapy serves as an indicator of response [247].

Future immunotherapy strategies in OC may rely on pretreatment biomarker-driven selection. A patient with a TIL-high, PD-L1-positive, interferon-gamma-rich tumor may proceed directly to combination checkpoint blockade, whereas a patient with a non-inflamed tumor might first receive an oncolytic virus or an epigenetic modifier such as an HDAC inhibitor to prime the tumor for immunotherapy [65]. As clinical trial data accumulate, biomarker refinement will improve patient stratification.

Advanced computational tools, such as artificial intelligence (AI) applied to pathology slides, could enhance biomarker discovery by identifying immune infiltration patterns that correlate with response [248]. Similarly, AI-driven analysis of sequencing data may refine neoantigen prediction models, improving the selection of targeted immunotherapies [249]. These developments hold the potential to personalize immunotherapy for OC, ensuring that only those patients most likely to respond receive immune-based treatments while others are directed toward alternative therapeutic strategies.

### 7.5. Challenge 5: Practical Deployment and Persistence of Cellular Therapies

Unlike conventional drug-based therapies, cell-based immunotherapies such as CAR-T and TIL therapy present significant manufacturing and logistical challenges that can hinder their clinical deployment in PROC. The process of generating autologous CAR-T cells requires extracting, expanding, and engineering a patient’s T cells [125,126], which can be particularly difficult if the patient is immunosuppressed from prior treatments. Similarly, TIL therapy necessitates surgical resection of tumor tissue, followed by ex vivo expansion of tumor-reactive T cells, requiring specialized manufacturing facilities and extended production times. These factors contribute to delays in treatment initiation, often spanning four to six weeks, during which tumor progression can occur, limiting the feasibility of cellular therapies for patients with rapidly advancing disease [250].

To address these challenges, off-the-shelf (allogeneic) cell therapies are being explored. Allogeneic CAR-T cells derived from healthy donors, with gene edits to prevent immune rejection, are being tested in some solid tumors, and similar approaches could be adapted for OC. A universal CAR-T targeting a common OC antigen, such as mesothelin, FRα, or MUC16, could provide a ready-to-use option without requiring patient-specific cell manufacturing [251]. For NK-cell therapies, allogeneic strategies are already being implemented [140]; CAR-NK cells derived from cord blood or NK cell lines offer a promising alternative that enables rapid administration and potential repeat dosing in cycles [138].

Beyond logistical constraints, cell persistence and durability of response remain critical challenges in solid tumors. Unlike CAR-T cells in hematologic malignancies, which can persist and expand in circulation, solid tumor CAR-T cells often display limited persistence due to T-cell exhaustion, immune rejection (if allogeneic), and the immunosuppressive TME. To improve longevity, next-generation cell products incorporate homeostatic cytokine support, such as membrane-bound IL-15, to enhance persistence and expansion in vivo [252]. Additionally, gene-edited T cells are being engineered to resist exhaustion by knocking out regulatory proteins such as diacylglycerol kinase, which modulates T-cell activation [253].

Innovative delivery strategies are also under investigation to sustain immune pressure on tumors. Future approaches may involve implantable “pipeline” devices that continuously release or activate immune cells within the peritoneal cavity, maintaining localized and sustained anti-tumor activity without requiring multiple infusions [254]. These advancements, alongside improvements in manufacturing efficiency and rapid deployment of cellular therapies, will be essential for translating adoptive cell therapies into widely accessible treatments for OC.

### 7.6. Challenge 6: Toxicity Management in Combinatorial Regimens

As immunotherapy combinations become more sophisticated in PROC, the challenge of managing toxicity grows increasingly important. Many OC patients are older, heavily pretreated with chemotherapy, and have reduced physiological reserves, making them particularly vulnerable to immune-related adverse events (irAEs). Dual checkpoint blockade, while potentially more effective, significantly increases the risk of autoimmune complications such as colitis, hepatitis, and endocrinopathies [255]. Similarly, bispecific TCEs and CAR-T therapies can induce CRS, which can be especially dangerous in frail patients with peritoneal ascites, causing fluid shifts and hemodynamic instability [256].

To mitigate toxicity, biomarker-driven monitoring and prophylactic interventions are being developed. Serum cytokine tracking may help identify patients at risk of severe immune reactions [257], while preemptive administration of IL-6 blockers (e.g., tocilizumab) during TCE therapy can prevent CRS [256]. Step-up dosing strategies, in which therapy is gradually escalated, are another approach to acclimate the immune system and reduce severe responses [258]. Beyond pharmacological interventions, rational drug design is shifting toward tumor-localized immunotherapy to minimize systemic exposure. Oncolytic viruses, for instance, are engineered to replicate selectively within tumors, ensuring that immune activation remains confined to the TME [259]. Similarly, IP delivery of bispecifics instead of IV administration concentrates the therapy within the peritoneal cavity, reducing systemic toxicity while enhancing efficacy [233].

For cell therapies, safety mechanisms such as suicide switches are being incorporated to allow rapid control over toxicity events. Some engineered T-cell products now include inducible caspase-9, which can be activated if severe toxicity occurs, leading to controlled elimination of the infused immune cells [260]. These innovations aim to push the boundaries of aggressive combinatorial immunotherapies while maintaining patient safety.

Looking forward, next-generation immunotherapy strategies will likely involve multi-modality combinations designed to enhance efficacy while minimizing toxicity. Clinical trials are already exploring convergent strategies, such as combining vaccines to prime T cells, CAR-T therapy for direct cytotoxicity, and bispecific checkpoint inhibitors (e.g., PD-1/LAG-3), to sustain immune responses. Industry collaborations, such as Turnstone’s oncolytic virus with AbbVie’s anti-PD-1 inhibitors [261] or Iovance’s TIL therapy with pembrolizumab [262], reflect the increasing interest in testing such synergistic approaches. Adaptive trial designs may further refine combination regimens, using real-time biomarker data (e.g., post-treatment biopsies showing PD-L1 expression) to dynamically adjust patient treatments.

Beyond treating active disease, immunotherapy may play a role in preventing platinum resistance or delaying recurrence. Early-intervention trials, such as JAVELIN OVARIAN 100 (NCT02718417) and 200 (NCT02580058), are testing checkpoint inhibitors as maintenance therapy following frontline chemotherapy to determine if the immune system can suppress microscopic residual disease before relapse occurs. Similarly, cancer vaccines could be used prophylactically in remission to train the immune system to eliminate recurrent disease at inception [198]. In the long term, prophylactic vaccines for high-risk individuals (e.g., *BRCA* mutation carriers) could even help reduce OC incidence, with platforms like OvarianVax in early development to target precursor lesions before malignant transformation [188].

The integration of omics and precision medicine will be crucial in tailoring immunotherapy regimens to individual patients. Genomic profiling could identify neoantigens or immune escape pathways, while proteomic analysis of ascites fluid may reveal cytokine and checkpoint signatures unique to each patient, guiding the selection of immune-modulating agents. Emerging cell therapy platforms are also exploring alternative immune cell types, such as gamma-delta T cells [148], invariant NKT cells, and macrophage-based therapies, each with unique tumor-killing mechanisms. For example, invariant NKT cells, which recognize lipid antigens via CD1d, are being investigated in combination with checkpoint blockade and CD1d-binding ligands (e.g., IMM60) to stimulate both innate and adaptive immunity [263].

Additionally, recent studies suggest that gut microbiome composition influences immunotherapy response in cancer patients. Specific probiotic or antibiotic regimens may either enhance or impair immune checkpoint blockade efficacy [227], making microbiome modulation another potential strategy for improving immunotherapy outcomes in PROC. Future trials may incorporate microbiome profiling to optimize patient selection and treatment customization.

As immunotherapy continues to evolve, rational combination regimens, precision-based patient selection, and toxicity management strategies will be essential in maximizing efficacy while ensuring safety in PROC.

## 8. Conclusions

The emergence of immunotherapy is transforming the treatment landscape for PROC, offering new hope where conventional therapies have failed. Advances since 2021, including cellular immunotherapies, bispecific antibodies, checkpoint inhibitors, oncolytic viruses, and therapeutic vaccines, demonstrate that even chemotherapy-refractory tumors can sometimes be controlled or regressed. The future of PROC treatment lies in rational combination strategies, integrating immune-based therapies with biomarkers to personalize treatment approaches.

While challenges remain, including immune evasion, antigen heterogeneity, and toxicity management, ongoing clinical trials—such as NCT05316129 (CAR-T), NCT05922930 (CAR-NK), NCT05281471 (oncolytic virus), and NCT03554649 (DC vaccine)—are generating critical insights that will optimize immunotherapy regimens. A comprehensive list of completed and ongoing clinical trials is provided in Appendix A. Innovative bioengineering approaches, such as multi-targeted CAR-T cells, engineered macrophages, and precision-guided bispecifics, are addressing tumor resistance mechanisms and improving immune cell persistence.

With academic research, biotechnology innovation, and strategic industry collaborations driving rapid progress, the next decade could see immunotherapy become an integral part of standard care for PROC. As the field continues to evolve, the goal is clear: to transform PROC from an untreatable condition into one where durable remission becomes a reality for more patients.

## Figures and Tables

**Table 1:** Therapeutic strategies for ROC.

Treatment Phase	Modality	Patient Subgroup	Examples
Surgical Options	Cytoreduction	PSROC (platinum-sensitive, resectable) ^1^	Secondary cytoreduction; palliative surgery for symptom relief
Chemotherapy	Platinum-based	PSROC	Carboplatin + paclitaxel, gemcitabine, PLD
	Non-platinum	PROC or platinum-refractory ROC	Weekly paclitaxel, PLD, gemcitabine, topotecan
Targeted Therapy	Anti-VEGF ^2^	General population (regardless of HRD)	Bevacizumab + chemotherapy for PSROC/PROC
	FRα overexpression	PROC with folate receptor alpha (FRα^+^)	Mirvetuximab soravtansine (MIRV)
Hormonal Therapy	Hormone receptor-positive	ER^+^/PR^+^ ROC ^3^	Tamoxifen, letrozole, anastrozole
Maintenance Therapy	PARP inhibitors (PARPi)	*BRCA1/2* mutated (germline or somatic)	Olaparib, niraparib, rucaparib
	HRD-positive, *BRCA* wild-type	-	Niraparib, rucaparib (selected indications)
	HR-proficient (HRp)	No biomarker approval, limited benefit	Used with caution or in trial settings
Treatment Strategy	Based on PFI	PSROC (PFI ≥ 6 months)	Platinum rechallenge
		PROC (PFI < 6 months)	Non-platinum regimens; considered targeted/hormonal therapy

^1^ In selected PSROC cases (e.g., DESKTOP III trial). ^2^ Vascular endothelial growth factor. ^3^ Not FDA-/EMA-approved specifically for OC, but endorsed in guidelines (e.g., NCCN, ESMO) for ER^+^/PR^+^ (estrogen receptor- and progesterone receptor-positive) ROC.

## Data Availability

No new data were created or analyzed in this study.

## Abbreviations

The following abbreviations are used in this manuscript:

ACT	adoptive cell transfer
ADCC	antibody-dependent cellular cytotoxicity
AIs	aromatase inhibitors
AQPs	aquaporins
BER	base excision repair
CA-125	cancer antigen 125
CAR	chimeric antigen receptor
CAR-M	chimeric antigen receptor macrophages
CAR-T	chimeric antigen receptor T-cell/s
CCNE1	cell cycle regulator cyclin E1
CRS	cytokine release syndrome
CSF1R	CSF-1 receptor
ctDNA	circulating tumor DNA
CTLA-4	cytotoxic T-cell antigen 4
CTRs	copper transport proteins
DC(s)	dendritic cell(s)
DDR	DNA damage response
EMA	European Medicines Agency
EOC(s)	epithelial ovarian cancer(s)
ER	estrogen receptor
ESMO	European Society For Medical Oncology
FDA	Food and Drug Administration
FMT	fecal microbiota transplantation
FRα	folate receptor alpha
FRβ	folate receptor beta
FSHR	follicle-stimulating hormone receptor
GVHD	graft-versus-host disease
HER2	human epidermal growth factor receptor 2
HGSC(s)	high-grade serous carcinoma(s)
HRD	homologous recombination deficiency; homologous recombination-deficient
HR	homologous repair
ICIs	immune checkpoint inhibitors
IFN-α	interferon-alpha
IL-2	interleukin-2
IP	intraperitoneal
irAEs	immune-related adverse events
IV	intravenous
LGSC(s)	low-grade serous carcinoma(s)
MDRs	multidrug resistance proteins
MDSCs	myeloid-derived suppressor cells
MHC	major histocompatibility complex
MIRV	Mirvetuximab soravtansine-gynx
MMR	mismatch repair
MSI-H	microsatellite instability-high
NCCN	National Comprehensive Cancer Network
NER	nucleotide excision repair
NHEJ	non-homologous end joining
NK	natural killer
OC(s)	ovarian cancer(s)
OCDC	oxidized autologous whole-tumor lysates
Olvi-Vec	Olvimulogene nanivacirepvec
ORR(s)	objective response rate(s)
OS	overall survival
PARPi	poly (ADP-ribose) polymerase inhibitors
PD-1	programmed death-1
PD-L1	programmed death-ligand 1
PFI	platinum-free interval
PFS	progression-free survival
PLD	pegylated liposomal doxorubicin
PPROC	primary platinum-resistant ovarian cancer
PROC	platinum-resistant ovarian cancer
PR	progesterone receptor
PSROC	platinum-sensitive recurrent ovarian cancer
RB1	retinoblastoma protein 1
ROC	recurrent ovarian cancer
ROS	reactive oxygen species
scFvs	single-chain variable fragments
SLCs	solute carrier transporters
SPROC	secondary platinum-resistant ovarian cancer
TAA(s)	tumor-associated antigen(s)
TAMs	tumor-associated macrophages
TCEs	T-cell engagers
TCR	T-cell receptor
TIL(s)	tumor-infiltrating lymphocyte(s)
TLR	Toll-like receptor
TME(s)	tumor microenvironment(s)
Tregs	regulatory T cells
TRICOM	Triad of Co-stimulatory Molecules
T-VEC	talimogene laherparepvec
VEGF	vascular endothelial growth factor

## Appendix A

**Table A1:** Selected clinical trials investigating immunotherapy approaches in OC.

Trial Name	Phase	Therapy Type	Agents Involved	Patient Population	Key Findings
IMagyn050	III	Checkpoint Inhibitor + Chemo	Atezolizumab + bevacizumab + chemo	Frontline OC	No significant PFS benefit in unselected patients
JAVELIN Ovarian 100	III	Checkpoint Inhibitor + Chemo	Avelumab + chemo	Frontline OC	No PFS or OS improvement
JAVELIN Ovarian 200	III	Checkpoint Inhibitor vs. Chemo	Avelumab vs. PLD	PROC	No significant survival benefit
KEYNOTE-B96	III	Checkpoint Inhibitor + Chemo	Pembrolizumab + chemo	PROC (PD-L1^+^ and all-comers)	Significantly improved PFS vs. chemo alone
KEYNOTE-100	II	ICI monotherapy	Pembrolizumab	Recurrent OC	Low ORR (~8%), better in PD-L1^+^ tumors
DUO-O	III	ICI + PARPi + Chemo	Durvalumab + olaparib + bevacizumab + chemo	Frontline OC	PFS improved in HRD^+^, no OS benefit yet
ATHENA	III	PARPi ± ICI	Rucaparib ± nivolumab	Frontline OC	Rucaparib improved PFS
ROSELLA	II	GR modulator	Relacorilant	PROC	Improved PFS when combined with nab-paclitaxel
DESKTOP III	III	Surgery	Secondary cytoreduction	PSROC	Improved OS in selected patients
NRG-GY003	II	Dual ICI	Nivolumab + ipilimumab	ROC	Better PFS and ORR vs. nivolumab alone
MEDIOLA	II	PARPi + ICI	Durvalumab + olaparib	*BRCA*-mutant OC	Encouraging ORR and PFS
TOPACIO/ KEYNOTE-162	I/II	PARPi + ICI	Niraparib + pembrolizumab	PROC	Clinical activity in *BRCA*^wt^/HRD^+^ tumors
MOONSTONE	III	PARPi + ICI	Niraparib + dostarlimab	PROC	Ongoing
ARTISTRY-7	III	IL-2 agonist ± ICI	Nemvaleukin ± pembrolizumab	PROC	Phase II data showed 28.6% ORR
OVERVEIL	III	Oncolytic virus + chemo	Olvi-Vec + chemo	PROC	Improved immune activation
City of Hope CAR-T	I	CAR-T	Anti-TAG72 CAR-T	OC	Ongoing
BNT211	I/II	CAR-T	CLDN6-targeted CAR-T	Solid tumors incl. OC	Early efficacy signals
LN-145	II	TIL therapy	LN-145	PROC	Under evaluation
CT-0508	I	CAR-M	HER2-directed CAR-M	Solid tumors incl. OC	Ongoing
SY001	I	CAR-M	Mesothelin-targeting CAR-M	Recurrent OC	First-in-human report in 2024
FLORA-5	II	ICI combo	Nivolumab + relatlimab	OC	Testing PD-1 + LAG-3 blockade
Tebotelimab trial	I	Bispecific Ab	PD-1 + LAG-3 bsAb	Solid tumors incl. OC	Enhanced T-cell activation
NCT04137900	I	ICI combo	TIM-3/TIGIT + PD-1 blockade	Advanced gynecologic cancers	Ongoing
XB002	I	ADC	B7-H4 targeting ADC	PROC	Early clinical trial
SL-172154	I	Bispecific	CD47 blockade + CD40 activation	PROC	Ongoing
GEN-1	I/II	Gene therapy	IL-12 nanoparticles	PROC	Local immune activation
